# Supplementary material for: Efficacy and safety of paravertebral block versus intercostal nerve block in thoracic surgery and breast surgery: A systematic review and meta-analysis
Source: PLoS One. 2020 Oct 5;15(10):e0237363. doi: 10.1371/journal.pone.0237363 (PMC7535861; doi:10.1371/journal.pone.0237363)
Supplement: S1 Appendix — (DOCX) [file pone.0237363.s001.docx]

**Search strategy**

Databases of **PubMed**, **Embase**, **Web of Science databases** and **Cochrane Library** were searched for relevant publications with the following Medical Subject Headings and search terms: **“paravertebral block”, “intercostal nerve block”,“Thoracic surgery”, “Thoracoscopic surgery” “thoracotomy” “Mastectomy” “breast surgery”**. The initial search was conducted in Feburary 9, 2020 without language restrictions. Anyway, we used the search phrases **((paravertebral block) AND intercostal nerve block) AND (Thoracic surgery or Thoracoscopic surgery or thoracotomy or Mastectomy or breast surgery)** to research and identify relevant articles.
