# Supplementary material for: Efficacy and safety of paravertebral block versus intercostal nerve block in thoracic surgery and breast surgery: A systematic review and meta-analysis
Source: PLoS One. 2020 Oct 5;15(10):e0237363. doi: 10.1371/journal.pone.0237363 (PMC7535861; doi:10.1371/journal.pone.0237363)
Supplement: S2 Appendix — (DOCX) [file pone.0237363.s002.docx]

**The results of the Egger's test**


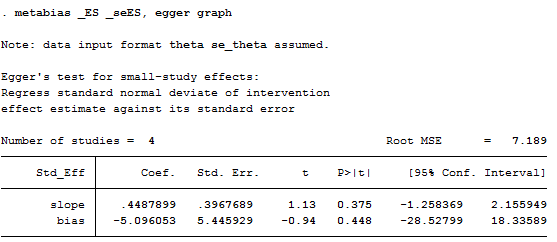


Publication bias of comparison: PVB vs INB, Outcome: VAS scores at rest at the first 1 h


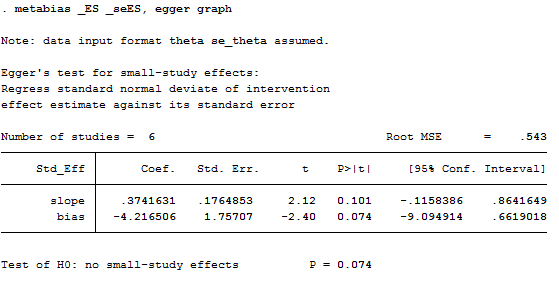


Publication bias of comparison: PVB vs INB, Outcome: VAS scores at rest at the first 2 h


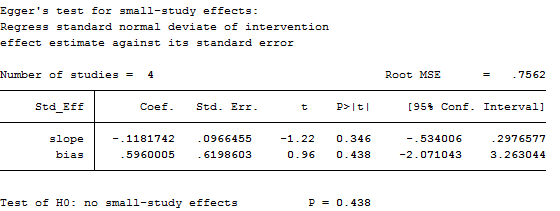


Publication bias of comparison: PVB vs INB, Outcome: VAS scores at rest at the first 12 h


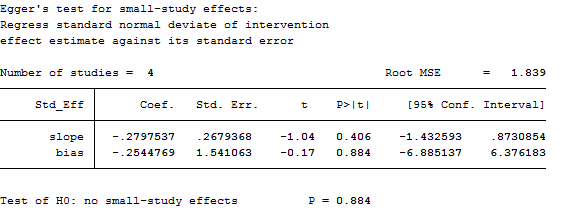


Publication bias of comparison: PVB vs INB, Outcome: VAS scores at rest at the first 24 h


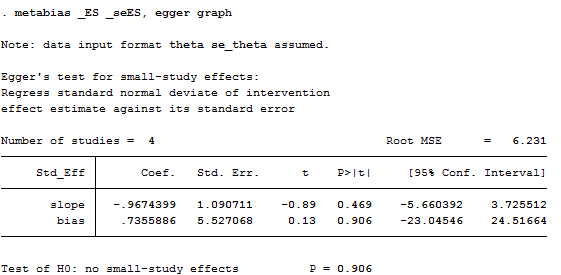


Publication bias of comparison: PVB vs INB, Outcome: rates of postoperative nausea and vomiting


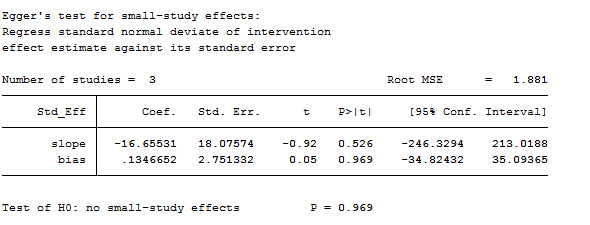


Publication bias of comparison: PVB vs INB, Outcome: consumption of postoperative morphine
